# Liver‐related long‐term outcomes of thiazolidinedione use in persons with type 2 diabetes

**DOI:** 10.1111/liv.14385

**Published:** 2020-02-10

**Authors:** Fu‐Shun Yen, Yu‐Cih Yang, Chii‐Min Hwu, James C.‐C. Wei, Yi‐Hsiang Huang, Ming‐Chih Hou, Chih‐Cheng Hsu

**Affiliations:** ^1^ Dr. Yen's Clinic Taoyuan City Taiwan; ^2^ Management Office for Health Data China Medical University Hospital Taichung Taiwan; ^3^ College of Medicine China Medical University Taichung Taiwan; ^4^ Faculty of Medicine National Yang‐Ming University School of Medicine Taipei Taiwan; ^5^ Section of Endocrinology and Metabolism Department of Medicine Taipei Veterans General Hospital Taipei Taiwan; ^6^ Division of Allergy Immunology and Rheumatology Chung Shan Medical University Hospital Taichung Taiwan; ^7^ Division of Gastroenterology and Hepatology Department of Medicine Taipei Veterans General Hospital Taipei Taiwan; ^8^ Institute of Clinical Medicine School of Medicine National Yang‐Ming University Taipei Taiwan; ^9^ Institute of Population Health Sciences National Health Research Institutes Zhunan Taiwan; ^10^ Department of Health Services Administration China Medical University Taichung Taiwan; ^11^ Department of Family Medicine Min‐Sheng General Hospital Taoyuan Taiwan

**Keywords:** nonalcoholic fatty liver disease, cirrhosis, hepatic decompensation, hepatic failure, all‐cause mortality, liver‐related death

## Abstract

**Background & Aims:**

Studies have described prominent histologic improvement in patients with nonalcoholic steatohepatitis (NASH) using thiazolidinedione (TZD); however, these were all short term with moderate sample size, no liver‐related long‐term outcomes could be noted.

**Methods:**

This retrospective cohort study enrolled patients with newly diagnosed type 2 diabetes mellitus (T2DM) from Taiwan's National Health Insurance Research Database between 1 January 2000 and 31 December 2013. We matched TZD users and nonusers at a 1:1 ratio through propensity score matching. This study included 5095 paired TZD users and nonusers. Cox proportional hazard models were used to compare the risks of cirrhosis, hepatic decompensation, hepatic failure and all‐cause mortality between TZD users and nonusers. The Kaplan‐Meier method was used to compare the cumulative incidence of these main outcomes.

**Results:**

The incidence rates of cirrhosis, hepatic decompensation, hepatic failure and all‐cause mortality during follow‐up were 0.77 vs 1.95, 1.43 vs 1.75, 0.36 vs 0.70, and 4.89 vs 3.78 per 1000 person‐years between TZD users and nonusers. The adjusted hazard ratios of cirrhosis, hepatic decompensation, hepatic failure and all‐cause mortality were 0.39 (95% confidence interval [CI]: 0.21‐0.72), 0.86 (95% CI: 0.52‐1.44), 0.46 (95% CI: 0.18‐1.17) and 1.18 (95% CI: 0.87‐1.61) respectively.

**Conclusions:**

Our study demonstrated that TZD use could significantly lower the risk of cirrhosis. In clinical settings, TZD use might be able to improve liver‐related long‐term outcomes.

**Abbreviations**
CCI: Charlson comorbidity index
CI: confidence interval
CV: cardiovascular
DCSI: Diabetes Complications Severity Index
DM: diabetes mellitus
HBV: hepatic B virus
HCC: hepatocellular carcinoma
HCV: hepatic C virus
HF: heart failure
HRs: hazard ratios
NAFLD: nonalcoholic fatty liver disease
NASH: nonalcoholic steatohepatitis
OADs: oral antidiabetic drugs
PPARγ: peroxisome proliferator‐activated receptor gamma
T2DM: type 2 diabetes mellitus
TZD: thiazolidinedione

**Key points**

The prevalence of T2DM and NAFLD has dramatically increased worldwide. Our study disclosed that TZD use could significantly lower the risk of cirrhosis as compared with no use. TZD use in patients with T2DM might improve their liver‐related long‐term outcomes.

## 1 INTRODUCTION

Owing to a sedentary lifestyle and westernized diet, the prevalence of type 2 diabetes mellitus (T2DM) and nonalcoholic fatty liver disease (NAFLD) has dramatically increased worldwide. According the IDF diabetes atlas, globally, diabetes mellitus (DM) cases increased from 151 million in 2000 to 425 million in 2017, representing an approximately 2.8‐fold increase in 17 years.^1^ In Taiwan, DM cases also increased from 707 000 in 2000^2^ to 1 958 000 in 2017, representing an approximately 2.77‐fold increase in 17 years. NAFLD is a new epidemic and is the most common cause of chronic liver disease^3^; its estimated worldwide prevalence is approximately 15%‐30%.^4^ People with T2DM frequently have dyslipidaemia and NAFLD. Approximately 40%‐70% of patients with T2DM have NAFLD^5^, ^6^; in Taiwan, approximately 43.3% of T2DM have NAFLD.^7^ NAFLD can progress to nonalcoholic steatohepatitis (NASH), hepatic fibrosis, cirrhosis and even hepatocellular carcinoma (HCC)^8^; it can also aggravate cardiovascular events in patients with T2DM.^9^ Furthermore, in patients with NAFLD, diabetes can increase the risks of hepatic complications and death.^10^

Thiazolidinediones (TZDs) are one of the most promising medications for treating NAFLD; studies have revealed histological improvement in patients with NASH, and fibrosis was even attenuated in some patients.^11^, ^12^, ^13^, ^14^, ^15^ TZDs bind and activate the nuclear receptor of peroxisome proliferator‐activated receptor gamma (PPARγ) with strong insulin‐sensitizing activity. They ameliorate insulin resistance by acting on adipose tissue, muscle and liver to increase glucose utilization and decrease glucose production. They can also increase adiponectin levels, reduce free fatty acid influx, increase fatty acid oxidation, and then decrease liver fat and attenuate hepatic inflammation.^16^

But until now, most of the TZD studies in patients with NAFLD or NASH are short term with few number of participants, no long‐term liver outcomes were noted. Therefore, we performed this nationwide cohort study to evaluate the liver‐related outcomes of TZD use in persons with type 2 diabetes.

## 2 METHODS

### 2.1 Study design and patients

In Taiwan, the National Health Insurance (NHI) programme has been implemented since 1995. At least 99% of the 23.5 million population of Taiwan are registered in this insurance programme.^17^ The National Health Insurance Research Database (NHIRD) includes the healthcare data of the insurants of the NHI programme, including sex, date of birth, residency area, medical procedures, drug prescriptions and diagnosis according to the International Classification of Diseases, Ninth Revision, Clinical Modification (ICD‐9‐CM). The Longitudinal Health Insurance Database 2000 (LHID2000) contains all the original claims data of 1 million beneficiaries randomly sampled from all insurants of the NHI programme in 2000. This cohort study was conducted using the LHID2000. All information that could be used to identify individuals or care providers was encrypted. This study was approved by the Research Ethics Committee of China Medical University and Hospital (CMUH104‐REC2‐115) and was granted a waiver of informed consent.

We selected patients with type 2 diabetes (T2DM) diagnosis ascertained through the presence of the ICD‐9‐CM code 250.xx in at least two outpatient records over 1 year or in one inpatient record in the LHID2000 between 1 January 2000 and 31 December 2013. We excluded patients diagnosed with DM before 1 January 2000, to ensure that only incident T2DM cases were included. This study excluded individuals younger than 30 years or older than 80 years, having follow‐up less than 180 days and those diagnosed with type 1 diabetes (Table S1), hepatitis B virus (HBV) infection, hepatitis C virus (HCV) infection, alcoholism, dialysis and heart failure (HF). This study also excluded patients diagnosed with cirrhosis, oesophageal varices, hepatic ascites, hepatic encephalopathy, jaundice, hepatic failure and HCC before the index date or within 180 days after index date. The algorithm for the definitions of diabetes and cirrhosis based on ICD‐9 coding has been validated in previous studies.^18^, ^19^

### 2.2 Procedures

We defined the first date of TZD use by our patients as the index date. Those who had not used any TZD in the observation period were considered TZD nonusers. Each TZD nonuser was randomly assigned an index date according to the corresponding index date of a TZD user. The TZDs examined in this study included pioglitazone and rosiglitazone (Troglitazone and ciglitazone were not used in Taiwan). The covariates analysed in multivariable models included baseline demographics (we grouped the diagnoses of overweight, abnormal weight gain, and BMI 25‐29 as overweight; obesity, BMI 30‐39, obesity complicated pregnancy as obesity; severe obesity, BMI ≥ 40, and bariatric surgery status for obesity as severe obesity), comorbidities diagnosed 1 year before the index date, and medications including antidiabetic agents, antihypertensive drugs, statin and aspirin. We used the Charlson comorbidity index (CCI) to quantify patients’ comorbidity profiles^20^ and the Diabetes Complications Severity Index (DCSI) score^21^ to define the severity of diabetes. CCI and DCSI scores were calculated according to patients’ records in the NHIRD 1 year before the index date.

### 2.3 Liver‐related long‐term outcomes

Using ICD‐9‐CM codes in medical records, we assessed the incidence rates of cirrhosis, hepatic decompensation (the composite of oesophageal varices, ascites, hepatic encephalopathy and jaundice),^22^ oesophageal varices, abdominal ascites, hepatic encephalopathy, jaundice, hepatic failure and HCC to determine liver‐related long‐term outcomes. We did a sensitivity analysis by excluding patients diagnosed with cirrhosis, oesophageal varices, hepatic ascites, hepatic encephalopathy, jaundice, hepatic failure, HCC or death within 365 days after index date.

### 2.4 Statistical analyses

Propensity score matching was used to optimize comparability between TZD users and nonusers.^23^ The propensity score was estimated for every patient using a nonparsimonious multivariable logistic regression, with TZD use as the dependent variable. In all, 26 clinically relevant covariates were used as independent variables (Table 1) in the regression. The nearest‐neighbour algorithm was applied to construct matched pairs, assuming that a proportion of 0.995‐1.0 was perfect.

**Table 1:** Baseline characteristics of study population

Variable	Original population				Standardized difference^a^	PS‐matching population				Standardized difference^a^
	Type II DM with TZDs (n = 6420)		Type II DM without TZDs (n = 66 766)			Type II DM with TZDs (n = 5095)		Type II DM without TZDs (n = 5095)		
	N	%	N	%		N	%	N	%	
Gender										
Female	3133	48.8	36 613	54.8	0.121	2436	47.8	2393	46.9	0.017
Male	3287	51.2	30 153	45.2	0.121	2659	52.2	2702	53.1	0.017
Age at baseline, year										
Mean (SD)	59.7 (10.7)		55.8 (12.3)		0.338	59.0 (10.9)		59.0 (11.1)		0.001
Comorbidity										
Overweight	29	0.45	376	0.56	0.016	22	0.43	19	0.37	0.009
Obesity	190	2.96	1632	2.44	0.032	154	3.02	153	3.00	0.001
Severe obesity	25	0.39	143	0.21	0.032	20	0.39	13	0.26	0.024
CCI score										
0	4762	74.2	56 493	84.6	0.26	3895	76.5	3930	77.1	0.016
1	778	12.1	5667	8.49	0.12	597	11.7	576	11.3	0.013
≥2	880	13.7	4606	6.90	0.225	603	11.8	589	11.6	0.009
DCSI score										
0	5162	80.4	60 644	90.8	0.3	4135	81.2	4134	81.1	0.001
1	389	6.06	1982	2.97	0.149	314	6.16	340	6.67	0.021
≥2	869	13.5	4140	6.20	0.248	646	12.7	621	12.2	0.015
Medication										
Oral antidiabetic drugs										
0‐1	279	4.35	48 215	72.2	1.95	279	5.48	289	5.67	0.009
2	1120	17.4	11 432	17.1	0.009	1118	21.9	1240	24.3	0.057
≥3	5021	78.2	7119	10.6	1.853	3698	72.6	3566	69.9	0.057
Metformin	6182	96.3	23 556	35.3	1.679	4857	95.3	4875	95.7	0.017
Sulfonylurea	6012	93.6	19 934	29.8	1.74	4687	91.9	4669	91.6	0.013
DPP‐4 inhibitors	3607	56.2	4266	6.39	1.273	2417	47.4	2262	44.4	0.061
AGIs	3395	52.8	4779	7.16	1.151	2294	45.0	2160	42.4	0.053
Meglitinides	2168	33.7	3123	4.68	0.794	1415	27.8	1331	26.1	0.037
Insulin	3471	54.1	8255	12.3	0.987	2425	47.6	2307	45.3	0.046
Antihypertensive drugs										
0‐1	1303	20.3	24 723	37.0	0.377	1123	22.0	1112	21.8	0.005
2	861	13.4	10 319	15.5	0.058	689	13.5	718	14.1	0.016
≥3	4256	66.3	31 724	47.5	0.386	3283	64.5	3265	64.1	0.007
Statin	2811	43.8	12 848	19.2	0.548	2054	40.3	2033	39.9	0.008
Aspirin	2982	46.5	18 776	28.1	0.386	2213	43.4	2185	42.9	0.011
Follow‐up time, y										
Mean (SD)	3.53 (2.58)		4.91 (3.90)		0.416	3.82 (2.71)		3.90 (3.01)		0.027

Abbreviations: AGI, Alpha‐glucosidase inhibitors; CCI, Charlson comorbidity index; DCSI, Diabetes complications severity index; DPP‐4 inhibitors, dipeptidyl peptidase‐4 inhibitors; TZDs, Thiazolidinediones.

a A standardized mean difference of ≤0.10 indicates a negligible difference between the two cohorts.

Cox proportional hazard models were used to compare the outcomes between TZD users and nonusers. All analyses were conducted using an intention‐to‐treat approach in accordance with the initial TZD assignment, irrespective of subsequent changes to other antidiabetic medications. The results are expressed as hazard ratios (HRs) with 95% confidence intervals (CIs). To calculate the risk of mortality, we censored patients at the time of death or the end of study, whichever occurred first. To calculate the risks of other investigated outcomes, we censored patients on the respective events or at the end of follow‐up on 31 December 2013, whichever occurred first. Using the Kaplan‐Meier method, we compared the cumulative incidence of cirrhosis over time between TZD users and nonusers.

We performed subgroup analysis according to pre‐specified strata of clinical interest to assess effect modification. The subgroup strata included overall rosiglitazone and pioglitazone use; sex; CCI score; oral antidiabetic drugs; insulin; antihypertensive drugs and statin. We calculate the *P* for interaction to see the different effects of variables in the same subgroup.

A two‐tailed *P* value less than .05 was considered significant. SAS version 9.2 was used for analyses.

## 3 RESULTS

### 3.1 Patients

**Figure 1:**
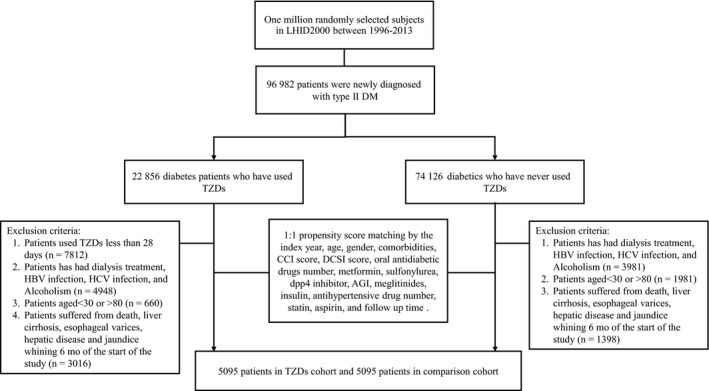
Flow chart of patient selection for this cohort study

We identified 22 856 patients newly diagnosed type 2 diabetes who used TZD and 74 126 patients newly diagnosed T2DM who had never used TZD between 1 January 2000 and 31 December 2013. The flowchart for patient selection for this study is depicted in Figure 1. After propensity score matching, 5095 pairs of patients were selected. The matched pairs were similar in terms of all covariates. The mean age of the cohort was 59.0 years, 52.7% of patients were men. The mean follow‐up time (mean [standard deviations, SD]) of the TZD users and non‐TZD users were 3.84 (2.71) and 3.90 (3.01) years respectively (Table 1).

### 3.2 Main outcomes

**Figure 2:**
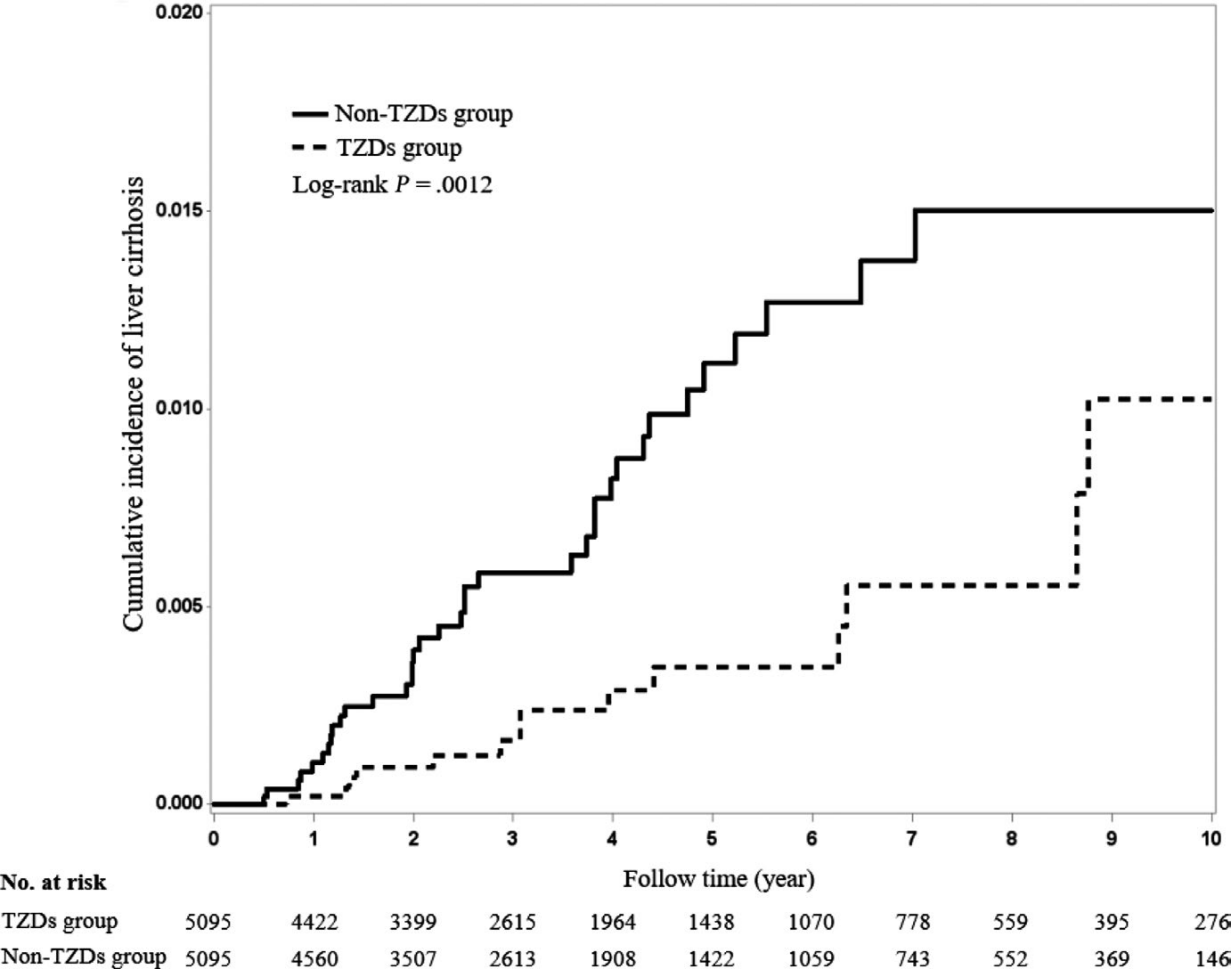
Cumulative incidence of cirrhosis between Thiazolidinediones users and nonusers in T2DM through Kaplan‐Meier

In this study, 96 (1.88%) TZD users and 76 (1.49%) nonusers died during the follow‐up period (incidence rate: 4.89 vs 3.78 per 1000 person‐years). Multivariable models showed that TZD users had no significant difference of mortality (aHR: 1.18, *P* = .27; Table 2). For liver‐related outcomes, TZD users appeared to have lower risks of cirrhosis (incidence rate: 0.77 vs 1.95 per 1000 person‐years; aHR: 0.39, *P* = .002; Table 2); TZD users appeared to have no significant difference in the risks of hepatic decompensation (aHR: 0.86, *P* = .58), hepatic failure (aHR: 0.46, *P* = .10) and HCC (aHR:1.22, *P* = .35) during the follow‐up period. Figure 2 delineates the cumulative incidence rates of cirrhosis between TZD users and nonusers, which were determined using the Kaplan‐Meier method.

**Table 2:** TZD users vs. nonusers in patients with type 2 diabetes after propensity matching

Outcome	TZDs user			TZDs nonuser			Crude		Multivariable adjusted	
	Event	PY	IR	Event	PY	IR	HR (95% CI)	*P* value	HR (95% CI)	*P* value
All‐cause mortality	96	19 621	4.89	76	20 124	3.78	1.30 (0.96‐1.76)	.08	1.18 (0.87‐1.61)	.27
Cirrhosis	15	19 565	0.77	39	20 023	1.95	0.41 (0.22‐0.75)	.004	0.39 (0.21‐0.72)	.002
Hepatic decompensation	28	19 533	1.43	35	20 037	1.75	0.89 (0.53‐1.47)	.65	0.86 (0.52‐1.44)	.58
Oesophageal varices	3	19 603	0.15	3	20 117	0.15	0.98 (0.19‐4.88)	.98	1.11 (0.22‐5.62)	.89
Hepatic ascites	9	19 613	0.46	13	20 110	0.65	0.74 (0.31‐1.77)	.50	0.73 (0.30‐1.75)	.48
Hepatic encephalopathy	1	196 161	0.01	0	20 121	0	—	—	—	—
Jaundice	10	19 584	0.51	13	20 093	0.65	0.77 (0.34‐1.77)	.55	0.74 (0.32‐1.70)	.48
Hepatic failure	7	19 607	0.36	14	20 056	0.70	0.51 (0.20‐1.27)	.15	0.46 (0.18‐1.17)	.10
Hepatic carcinoma	21	19 582	1.07	23	20 085	1.15	1.08 (0.97‐3.49)	.09	1.22 (0.95‐2.49)	.35

Note

Decompensated cirrhosis contains oesophageal varices, hepatic ascites, hepatic encephalopathy, hepatic Jaundice.

HR adjusted for gender, age, comorbidities, CCI score, DCSI score and medications use.

— Unable to calculate because there are few or no events in with and without TZD cohorts.

Abbreviations: CI, confidence interval; HR, hazard ratio; IR, incidence rate, per 1000 person‐years; PY, person‐years; TZDs, Thiazolidinediones.

### 3.3 Subgroup analysis

Table 3 presents the results of subgroup analysis of cirrhosis between TZD users and nonusers. Compared with TZD nonusers, rosiglitazone and pioglitazone users, men, patients undergoing insulin treatment, using ≧3 oral antidiabetic drugs, and statin nonusers had a significantly lower risk of cirrhosis. We used the p for interaction to compare rosiglitazone and pioglitazone on the effect of cirrhosis, which show no significant difference (*P* = .9209).

**Table 3:** Incidence and Cox proportional hazard regression with hazard ratios and 95% confidence intervals of cirrhosis associated with and without TZD by gender, age group and comorbidities

Variable	TZD						Adjusted HR (95% CI)	*P* for interaction
	No			Yes				
	Event	Person‐year	IR	Event	Person‐year	IR		
Overall	39	20 023	1.95	15	19 565	0.77	0.39 (0.21‐0.72)**	
Rosiglitazone	39	20 023	1.95	6	8934	0.67	0.43 (0.20‐0.90)*	.9209
Pioglitazone	39	20 023	1.95	9	10 631	0.84	0.35 (0.15‐0.85)*	
Gender								
Female	15	9604	1.56	6	9560	0.63	0.72 (0.25‐2.00)	.1648
Male	24	10 419	2.3	9	10 005	0.9	0.31 (0.10‐0.92)*	
CCI index								
0	30	15 882	1.89	13	15 027	0.87	0.46 (0.20‐1.08)	.7319
1	6	2137	2.81	1	2318	0.43	0.22 (0.02‐1.97)	
≥2	3	2004	1.5	1	2220	0.45	0.13 (0.007‐2.65)	
OAD								
0‐1	0	1006	0	1	975	1.03	—	.3839
2	10	4122	2.43	4	4119	0.97	0.31 (0.06‐1.51)	
≥3	29	14 895	1.95	10	14 471	0.69	0.40 (0.16‐0.98)*	
Insulin								
No	11	10 047	1.09	3	9305	0.32	0.52 (0.14‐1.90)	.0017
Yes	28	9976	2.81	12	10 260	1.17	0.39 (0.16‐0.97)*	
Antihypertensive drugs								
0‐1	2	3811	0.52	1	3950	0.25	—	.0588
2	8	2609	3.07	1	2517	0.4	0.24 (0.02‐1.97)	
≥3	29	13 603	2.13	13	13 098	0.99	0.51 (0.23‐1.14)	
Statin								
No	27	11 410	2.37	8	11 255	0.71	0.41 (0.16‐0.99)*	.2356
Yes	12	8613	1.39	7	8310	0.84	0.45 (0.11‐1.71)	

Note

HR adjusted for gender, age, comorbidities, CCI score, DCSI score, and medications use.

— Unable to calculate because there are few or no events in with and without TZD cohorts.

Abbreviations: CCI, Charlson comorbidity index; CI, confidence interval; HR, hazard ratio; IR, incidence rate, per 1000 person‐years; OAD, oral antidiabetic drugs; PY, person‐years; TZDs, Thiazolidinediones.

* *P* < .05.

** *P* < .01.

### 3.4 Sensitivity test

After excluding patients with liver‐related events or death within 365 days after index date, the adjusted HR of cirrhosis in TZD users was 0.37 (95% CI: 0.20‐0.65, *P* = .0006) compared with nonusers. TZD users had a significantly lower risk of cirrhosis (Tables S2 and S3).

## 4 DISCUSSION

Our study demonstrated that TZD use in T2DM could significantly decrease the risk of cirrhosis. Subgroup analysis revealed that both rosiglitazone and pioglitazone could lower the risks of cirrhosis with no significantly different effects between these two drugs.

Belfort et al randomly compared a hypocaloric diet plus pioglitazone with the diet plus placebo in 55 patients with impaired glucose tolerance or T2DM. The pioglitazone group showed reduced liver function and hepatic fat content, increased insulin sensitivity, and improved histologic steatosis, ballooning necrosis and inflammation but no significant difference in improvement of fibrosis compared with the placebo group.^11^ Cusi et al conducted a similar RCT to compare 101 pre‐diabetes or T2DM patients, consuming a hypocaloric diet plus pioglitazone or the diet plus placebo. The pioglitazone group showed reduced liver triglyceride content and improved histological scores of steatosis, inflammation, ballooning and fibrosis.^14^ Aithal et al compared the standard diet and exercise with pioglitazone or with placebo in 74 nondiabetic patients with NASH.^12^ The pioglitazone group showed reduced alanine aminotransferase levels and improved histologic features of hepatic injury, Mallory bodies and fibrosis compared with the placebo group. Sanyal et al conducted a RCT to compare vitamin E and pioglitazone with placebo in nondiabetic patients with NASH.^13^ Compared with placebo, both vitamin E and pioglitazone could reduce aminotransferase levels, decrease hepatic steatosis and lobular inflammation, but they could not improve fibrosis scores. Ratziu et al randomly assigned 63 patients with NASH to receive rosiglitazone or placebo treatment. The rosiglitazone group had improved 21% of steatosis and normalized 21% of transaminase levels. No improvement in ballooning, inflammation and fibrosis was noted.^15^ The systemic review and meta‐analysis of TZD use in patents with NASH has revealed that TZD could reduce liver fat, normalize aminotransferase levels and improve histological steatosis, ballooning and inflammation.^24^ These researches indicated that TZD use in patients with NASH could attenuate hepatic injury, inflammation and even fibrosis. However, these studies were all short‐term clinical trials with a moderate sample size; no large series study of long‐term liver‐related outcomes has been conducted.

To the best of our knowledge, our study is the first large series cohort study investigating liver‐related long‐term outcomes of TZD use in patients with T2DM. Though our patients were not image or histology confirmed NAFLD or NASH cases; however, based on epidemiological studies, at least 50% of patients with T2DM exhibit NAFLD.^5^ In addition, we excluded patients with previous viral hepatitis and alcoholism to make our population more similar to patients with NAFLD. This study indicated that TZD use in patients with T2DM might be able to prevent the development of cirrhosis; T2DM patients with the risk of hepatic injury could use TZD to prevent bad liver‐related long‐term outcomes.

Through the activation of PPARγ, TZD can reduce insulin resistance, sequester fatty acid in adipose tissue, and alleviate fat storage, steatosis and ballooning in the liver. TZDs also can activate AMP‐activated protein kinase (AMPK) and reduce hepatic fat content.^25^ TZD use can increase adiponectin and reduce high‐sensitivity C‐reactive protein (hs‐CRP), TNFα, IL‐1β and IL‐6 levels, which can reduce hepatic inflammation.^26^, ^27^ In preclinical studies, TZDs bind to PPARγ and thus inhibit the activation of hepatic stellate cells, reduce extracellular matrix production, decrease transforming growth factor β1 expression, attenuate matrix remodelling, and protect for tissue repair, fibrosis and even cirrhosis.^28^

TZD can induce cell cycle arrest and apoptosis and inhibit cancer cell proliferation and invasion through the activation of PPARγ.^29^ Some studies have revealed that TZD use may decrease the risk of HCC in T2DM.^30^, ^31^ However, another study provided contrasting results.^32^ A meta‐analysis disclosed that TZD does not decrease the risk of HCC,^33^ which is consistent with our result.

Our study has several strengths. We recruited patients from the NHIRD, which covers approximately 99% of the population of Taiwan. This might be able to decrease the risk of selection bias in the study. We used medical records instead of self‐reports, which might decrease recall bias and more correctly censor the incident rates of the main outcomes. The events noted within 6 months after the index date were excluded to decrease the possibility of latent morbidity and mortality. We did a sensitivity test to exclude patients with liver‐related events or death within 365 days after the index date, which also revealed that TZD could significantly lower the risk of cirrhosis.

Our study has some limitations. First, the NHIRD does not contain detailed information on patients’ lifestyle, height and body weight, and family history; all of which might influence the measured outcomes. Although we took many codings to include overweight, obesity and severe obesity as covariates in analysis, many patients’ obesity might not be recorded in the database, which could lead to the underestimation of the prevalence of obesity in our study. Second, the use of ICD‐9 codings for the censoring of cases in administrative databases has been criticized about its accuracy. In this study, the algorithm of using ICD‐9 to define type 2 diabetes and cirrhosis has been validated in the Taiwan National Health Insurance Research Database^18^ and in the administrative database from Parkland health and Hospital System,^19^ with acceptable accuracy. Using the NHI claim databases, we may avoid the measurement errors introduced by poor patient recall. Third, we lacked the results of biochemical tests and image examinations; therefore, we could not confirm the diagnosis of NAFLD in this dataset. This made us be difficult to observe the effects of TZD on NAFLD progression, which may be considered as intermediate processes in hepatic pathological changes. However, instead of the proxy indictor, we used several hard outcomes, including cirrhosis, hepatic failure, hepatic decompensation and mortality, to elucidate liver outcomes resulting from the TZD use. We believe important clinical implications can be derived from our study. Finally, our study was a cohort study with inevitable biases certainly existed. A larger randomized control study should be conducted to observe the liver‐specific endpoints after TZD use in patients with T2DM.

## 5 CONCLUSIONS

Our nationwide cohort study revealed that compared with TZD nonuse, TZD use in type 2 diabetes could significantly lower the risk of cirrhosis. In clinical settings, TZD use in T2DM patients might be able to improve their liver‐related long‐term outcomes.

## CONFLICT OF INTEREST

The authors do not have any disclosures to report.

## AUTHORS' CONTRIBUTIONS

*Specific author contributions*: Dr Ming‐Chih Hou and Chih‐Cheng Hsu had full access to all the data in the study and take responsibility for the integrity of the data and the accuracy of data analysis. Concept and design: Fu‐Shun Yen, James C.‐C. Wei and Yi‐Hsiang Huang; acquisition, analysis and interpretation of data: Fu‐Shun Yen and Chii‐Min Hwu; drafting of the manuscript: Fu‐Shun Yen, Ming‐Chih Hou and Chih‐Cheng Hsu; critical revision of the manuscript for important intellectual content: All authors; statistical analysis: Fu‐Shun Yen, Yu‐Cih Yang, Chii‐Min Hwu and Chih‐Cheng Hsu; obtained funding: Yu‐Cih Yang; administrative, technical and material support: Yu‐Cih Yang and James C.‐C. Wei; supervision: Ming‐Chih Hou and Chih‐Cheng Hsu.
