# Supplementary material for: Liver‐related long‐term outcomes of thiazolidinedione use in persons with type 2 diabetes
Source: Liver Int. 2020 Feb 10;40(5):1089–97. doi: 10.1111/liv.14385 (PMC7317545; doi:10.1111/liv.14385)
Supplement: Supplementary file 1 [file LIV-40-1089-s001.docx]

**Supporting Information**

**SUPPLEMENTARY TABLE 1** Diseases and related ICD-9-CM codes

| **Disease** | **ICD-9-CM codes** |
| --- | --- |
| Type 2 diabetes | 250.xx |
| Type 1 diabetes | 250.1x |
| Dialysis | 39.95, V56.0, V56.8, and V45.1 |
| Heart failure | 428 |
| Liver cirrhosis | 571.5, 571.2, and 571.6 |
| Esophageal varices | 456.0, 456.1, and 456.2 |
| Hepatic ascites | 789.59 |
| Hepatic encephalopathy | 572.2 |
| Jaundice | 782.4 |
| Hepatic failure | 570, 572.2, 572.4, and 572.8 |
| Hepatocellular carcinoma | 155.x |
| Overweight | 278.02 |
| Abnormal weight gain | 783.1 |
| Obesity | 278.00 and V77.8 |
| Obesity complicated pregnancy | 649.1 |
| Severe obesity | 278.01 |
| Bariatric surgery status for obesity | 649.2 and V45.86 |
| BMI 25-29 | V85.2 |
| BMI 30-39 | V85.3 |
| BMI ≧40 | V85.4 |
| Hepatic B virus infection | 070.2, 070.3, and V02.61 |
| Hepatic C virus infection | 070.41, 070.44, 070.51, 070.54, 070.70, 070.71, and V02.62 |
| Alcoholism | 291, 291.2, 291.4, 291.9, 303.0-303.9, 305.0, V11.3, V61.41, and V79.1 |

**SUPPLEMENTARY TABLE 2** Baseline characteristics of study population (excluding patients follow up less than 365 days)

| **Variable** | **Original population** | | | | | **Standardized difference §** | **PS-matching population** | | | | **Standardized difference §** |
| --- | --- | --- | --- | --- | --- | --- | --- | --- | --- | --- | --- |
|  | **Type II DM-with TZDs**  **(n=6285)** | | | **Type II DM-without TZDs**  **(n=58874)** | |  | **Type II DM-with TZDs**  **(n=5090)** | | **Type II DM-without TZDs**  **(n=5090)** | |  |
|  | **N** | | **%** | **N** | **%** |  | **N** | **%** | **N** | **%** |  |
| **Gender** |  | |  |  |  |  |  |  |  |  |  |
| female | 3072 | | 48.9 | 32591 | 55.4 | 0.13 | 2453 | 48.2 | 2376 | 46.7 | 0.03 |
| male | 3213 | | 51.1 | 26283 | 44.6 | 0.13 | 2637 | 51.8 | 2714 | 53.3 | 0.03 |
| **Age at baseline, year** |  | |  |  |  |  |  |  |  |  |  |
| mean(SD) | 59.4 (10.8) | | | 55.6 (12.4) | | 0.327 | 58.9 (10.9) | | 58.8 (11.1) | | 0.012 |
| **Comorbidity** |  | |  |  |  |  |  |  |  |  |  |
| Overweight | 29 | | 0.46 | 316 | 0.54 | 0.011 | 23 | 0.45 | 20 | 0.39 | 0.009 |
| Obesity | 200 | | 3.18 | 1400 | 2.38 | 0.049 | 152 | 2.99 | 148 | 2.91 | 0.005 |
| Severe obesity | 25 | | 0.40 | 122 | 0.21 | 0.035 | 21 | 0.41 | 16 | 0.31 | 0.016 |
| **CCI score** |  | |  |  |  |  |  |  |  |  |  |
| 0 | 4672 | | 74.3 | 50072 | 85.1 | 0.269 | 3896 | 76.5 | 3924 | 77.1 | 0.013 |
| 1 | 767 | | 12.2 | 4967 | 8.44 | 0.124 | 612 | 12.1 | 610 | 12.0 | 0.001 |
| ≥2 | 846 | | 13.5 | 3835 | 6.51 | 0.233 | 582 | 11.4 | 556 | 10.9 | 0.016 |
| **DCSI score** |  | |  |  |  |  |  |  |  |  |  |
| 0 | 5070 | | 80.7 | 54149 | 91.9 | 0.334 | 4164 | 81.8 | 4163 | 81.8 | 0.001 |
| 1 | 378 | | 6.01 | 1413 | 2.40 | 0.181 | 300 | 5.89 | 314 | 6.17 | 0.012 |
| ≥2 | 837 | | 13.3 | 3312 | 5.63 | 0.265 | 626 | 12.3 | 613 | 12.0 | 0.008 |
| **Medication** |  | |  |  |  |  |  |  |  |  |  |
| Oral antidiabetic drugs | |  |  |  |  |  |  |  |  |  |  |
| 0-1 | 302 | | 4.81 | 42408 | 72.0 | 1.912 | 302 | 5.93 | 307 | 6.03 | 0.004 |
| 2 | 1197 | | 19.1 | 9982 | 16.9 | 0.054 | 1197 | 23.5 | 1307 | 25.7 | 0.05 |
| ≥3 | 4786 | | 76.1 | 6484 | 11.0 | 1.742 | 3591 | 70.5 | 3476 | 68.3 | 0.049 |
| Metformin | 6034 | | 96.0 | 20497 | 34.8 | 1.68 | 4840 | 95.1 | 4854 | 95.3 | 0.013 |
| Sulfonylurea | 5869 | | 93.4 | 17680 | 30.0 | 1.718 | 4674 | 91.8 | 4685 | 92.0 | 0.008 |
| DPP-4 inhibitors | 3364 | | 53.5 | 3856 | 6.55 | 1.193 | 2283 | 44.8 | 2163 | 42.5 | 0.048 |
| AGIs | 3237 | | 51.5 | 4317 | 7.33 | 1.108 | 2239 | 43.9 | 2109 | 41.4 | 0.052 |
| Meglitinides | 2103 | | 33.5 | 2832 | 4.81 | 0.782 | 1392 | 27.3 | 1347 | 26.4 | 0.02 |
| Insulin | 3410 | | 54.2 | 7268 | 12.3 | 0.993 | 2446 | 48.0 | 2333 | 45.8 | 0.044 |
| Antihypertensive drugs | |  |  |  |  |  |  |  |  |  |  |
| 0-1 | 1284 | | 20.4 | 21680 | 36.8 | 0.369 | 1102 | 21.6 | 1103 | 21.7 | 0.001 |
| 2 | 840 | | 13.4 | 8990 | 15.3 | 0.054 | 691 | 13.6 | 702 | 13.8 | 0.006 |
| ≥3 | 4161 | | 66.2 | 28204 | 47.9 | 0.376 | 3297 | 64.8 | 3285 | 64.5 | 0.005 |
| Statin | 2712 | | 43.1 | 11496 | 19.5 | 0.527 | 2032 | 39.9 | 1981 | 38.9 | 0.021 |
| Aspirin | 2944 | | 46.8 | 16914 | 28.7 | 0.38 | 2268 | 44.6 | 2219 | 43.6 | 0.019 |
| **Follow up time, year** |  | |  |  |  |  |  |  |  |  |  |
| mean(SD) | 4.01 (2.57) | | | 5.46 (3.81) | | 0.446 | 4.28 (2.69) | | 4.28 (2.94) | | 0.001 |

TZDs, Thiazolidinediones; CCI, Charlson comorbidity index; DCSI, Diabetes complications severity index; DPP-4 inhibitors, dipeptidyl peptidase-4 inhibitors; AGI, Alpha-glucosidase inhibitors.

*P-value using chi-square for the comparisons between with and without TZDs.

§A standardized mean difference of ≤0.10 indicates a negligible difference between the two cohorts.

**SUPPLEMENTARY TABLE 3** TZD users vs. non-users in patients with type 2 diabetes after propensity matching (excluding patients follow up less than 365 days)

| **Outcome** | **TZDs user** | | | **TZDs non-user** | | | **Crude** | | **Multivariable adjusted** | |
| --- | --- | --- | --- | --- | --- | --- | --- | --- | --- | --- |
|  | **Event** | **PY** | **IR** | **Event** | **PY** | **IR** | **HR (95%CI)** | ***P* value** | **HR (95%CI)** | ***P* value** |
| All-cause mortality | 88 | 21914 | 4.01 | 85 | 22000 | 3.86 | 1.06 (0.78-1.43) | 0.69 | 0.97 (0.71-1.31) | 0.84 |
| Liver cirrhosis | 17 | 21864 | 0.77 | 41 | 21882 | 1.87 | 0.40 (0.23-0.71) | 0.001 | 0.37 (0.20-0.65) | 0.0006 |
| Hepatic decompensation | 28 | 21865 | 1.28 | 37 | 21944 | 1.68 | 0.74 (0.45-1.20) | 0.22 | 0.72 (0.44-1.18) | 0.19 |
| Esophageal varices | 3 | 21899 | 0.13 | 6 | 21982 | 0.27 | 0.49 (0.21-1.95) | 0.31 | 0.50 (0.12-2.04) | 0.34 |
| Hepatic ascites | 8 | 21906 | 0.36 | 10 | 21967 | 0.45 | 0.77 (0.30-1.97) | 0.28 | 0.76 (0.30-1.96) | 0.58 |
| Hepatic encephalopathy | 2 | 21914 | 0.09 | 2 | 21994 | 0.09 | 0.98 (0.13-6.99) | 0.98 | 0.84 (0.10-6.60) | 0.87 |
| Jaundice | 11 | 21887 | 0.50 | 11 | 21985 | 0.50 | 0.98 (0.42-2.28) | 0.97 | 0.96 (0.41-2.22) | 0.92 |
| Hepatic failure | 10 | 21898 | 0.45 | 12 | 21981 | 0.54 | 0.81 (0.35-1.88) | 0.63 | 0.80 (0.34-1.88) | 0.62 |

TZDs, Thiazolidinediones; PY, person-years; IR, incidence rate, per 1000 person-years; HR, hazard ratio; CI, confidence interval;

Decompensated cirrhosis contains esophageal varices, hepatic ascites, hepatic encephalopathy, hepatic Jaundice.

HR adjusted for gender, age, comorbidities, CCI score, DCSI score, and medications use.

*p<0.05, **p<0.01, ***p<0.00
